# High-intensity interval training improves metabolic syndrome and body composition in outpatient cardiac rehabilitation patients with myocardial infarction

**DOI:** 10.1186/s12933-019-0907-0

**Published:** 2019-08-14

**Authors:** Yaoshan Dun, Randal J. Thomas, Joshua R. Smith, Jose R. Medina-Inojosa, Ray W. Squires, Amanda R. Bonikowske, Hsuhang Huang, Suixin Liu, Thomas P. Olson

**Affiliations:** 1 Division of Cardiac Rehabilitation, Department of Physical Medicine & Rehabilitation, Xiangya Hospital Central South University, 87 Xiangya Road, Changsha, Hunan People’s Republic of China; 2 Division of Preventive Cardiology, Department of Cardiovascular Medicine, Mayo Clinic, 200 First Street SW, Rochester, MN 55905 USA

**Keywords:** Cardiac rehabilitation, High-intensity interval training, Metabolic syndrome, Myocardial infarction, Body composition

## Abstract

**Background:**

To examine the effect of high-intensity interval training (HIIT) on metabolic syndrome (MetS) and body composition in cardiac rehabilitation (CR) patients with myocardial infarction (MI).

**Methods:**

We retrospectively screened 174 consecutive patients with MetS enrolled in CR following MI between 2015 and 2018. We included 56 patients who completed 36 CR sessions and pre-post dual-energy X-ray absorptiometry. Of these patients, 42 engaged in HIIT and 14 in moderate-intensity continuous training (MICT). HIIT included 4–8 intervals of high-intensity (30–60 s at RPE 15–17 [Borg 6–20]) and low-intensity (1–5 min at RPE < 14), and MICT included 20–45 min of exercise at RPE 12–14. MetS and body composition variables were compared between MICT and HIIT groups.

**Results:**

Compared to MICT, HIIT demonstrated greater reductions in MetS (relative risk = 0.5, 95% CI 0.33–0.75, *P *< .001), MetS z-score (− 3.6 ± 2.9 vs. − 0.8 ± 3.8, *P *< .001) and improved MetS components: waist circumference (− 3 ± 5 vs. 1 ± 5 cm, *P *= .01), fasting blood glucose (− 25.8 ± 34.8 vs. − 3.9 ± 25.8 mg/dl, *P *< .001), triglycerides (− 67.8 ± 86.7 vs. − 10.4 ± 105.3 mg/dl, *P *< .001), and diastolic blood pressure (− 7 ± 11 vs. 0 ± 13 mmHg, *P *= .001). HIIT group demonstrated greater reductions in body fat mass (− 2.1 ± 2.1 vs. 0 ± 2.2 kg, *P *= .002), with increased body lean mass (0.9 ± 1.9 vs. − 0.9 ± 3.2 kg, *P *= .01) than the MICT. After matching for exercise energy expenditure, HIIT-induced improvements persisted for MetS z-score (*P *< .001), MetS components (*P *< .05), body fat mass (*P *= .002), body fat (*P *= .01), and lean mass (*P *= .03).

**Conclusions:**

Our data suggest that, compared to MICT, supervised HIIT results in greater improvements in MetS and body composition in MI patients with MetS undergoing CR.

## Background

Metabolic syndrome (MetS) is a constellation of cardiovascular risk factors that include abdominal adiposity, impaired glucose tolerance, hypertension, low serum high-density lipoprotein cholesterol and hypertriglyceridemia [1], and is associated with an eightfold increase in the risk of myocardial infarction (MI) [2]. Presence of MetS in patients with MI is associated with increased risk of adverse cardiovascular events and recurrent MI [3]. Obesity, especially abdominal adiposity is also considered an initial and principal pathophysiologic component of MetS [4]. More effective interventions to ameliorate MetS and reduce body fat among survivors of MI are critical to improving MI outcomes.

Current guidelines recommend exercise training as adjunctive therapy in patients with MI [5]. Exercise training improves body composition, cardiovascular, and metabolic outcomes in people with MetS [6]. High volume of physical activity is associated with lower rates of CVD events, glycosylated hemoglobin (HbA1C %), and systolic blood pressure in older adults [7]. However, universal agreement on the most effective exercise prescription to improve MetS for patients with MI remains unclear. Recent studies have demonstrated that high-intensity interval training (HIIT) may ameliorate MetS in obese adults [8], optimize HbA1C in patients with type II diabetes [9], and improve cardiac function in MI patients [10], more so than moderate-intensity continuous training (MICT) [8, 10]. However, the impact of HIIT on MetS in patients with MI undergoing early outpatient cardiac rehabilitation (CR) has not been studied.

The purpose of this study was to examine the effect of supervised HIIT on MetS presence and severity, and body composition in patients with MI undergoing early outpatient CR. We hypothesized that HIIT will be associated with greater improvements in MetS and body composition compared to MICT.

## Methods

### Design and participants

**Fig. 1:**
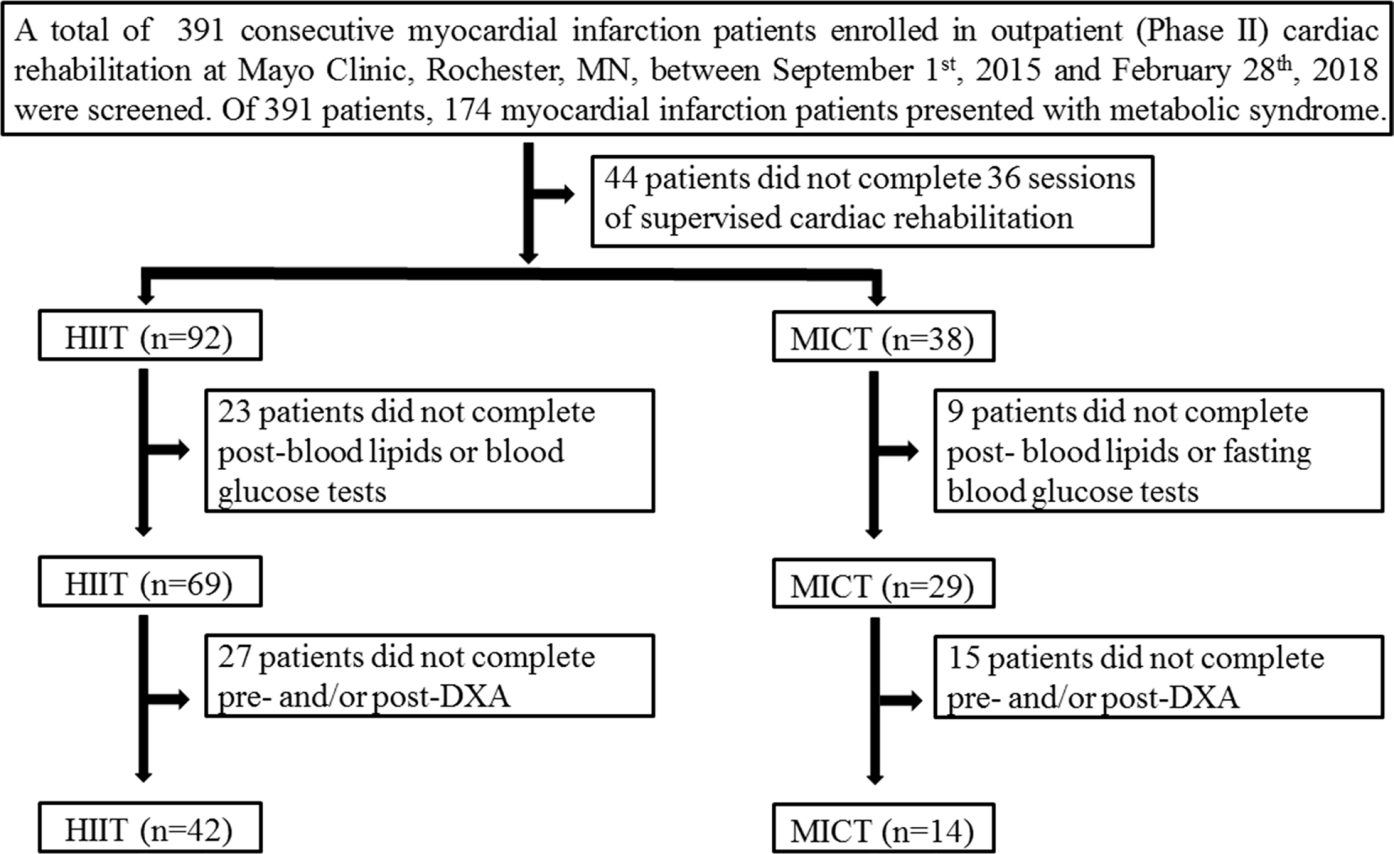
Study enrollment. *HIIT* high-intensity interval training, *MICT* moderate-intensity continuous training, *DXA* dual-energy X-ray absorptiometry

This was a retrospective cohort study of 391 consecutive MI patients enrolled in early outpatient cardiac rehabilitation (CR) at Mayo Clinic, Rochester, MN between September 1st, 2015 and February 28th, 2018. Of 391 patients, 174 MI patients presented with MetS. Patients meeting the following inclusion criteria were included: (1) diagnosed with both MI and MetS, (2) completion of 36 sessions of supervised outpatient cardiac rehabilitation, (3) pre- and post-CR blood lipids and fasting blood glucose tests, and (4) completed pre- and post-CR dual energy X-ray absorptiometry. The cardiac rehabilitation program at Mayo Clinic is a multidisciplinary health care program that includes a highly supervised exercise program (36 exercise sessions), patient education and wellness programs, nutrition and smoking cessation counseling, stress management, and psychological and pharmacological counseling. A standard 36 sessions of CR usually lasts 12 weeks (three sessions per week). Of the 56 eligible patients who met all the inclusion criteria, 42 engaged in HIIT and 14 in MICT (Fig. 1). All aspects of this study conformed to the principles outlined in the Declaration of Helsinki and were approved by the Mayo Clinic Institutional Review Board. All participants agreed to use of their medical records for research purposes.

### HIIT and MICT protocols

Patients performed an average of three supervised HIIT or MICT sessions/week on a treadmill, cycle ergometer, or recumbent stepper in the CR center and were prescribed 3 days of self-guided exercise at home. In the 1st week, all patients performed MICT, after that, if patients were able to exercise for ≥ 20 min at a rating of perceived exertion (RPE: Borg 6–20) of 12–14, they self-determined to continue MICT or transition to HIIT. The HIIT sessions were characterized by brief, intermittent bouts of high-intensity exercise interspersed with periods of low-intensity exercise (active recovery). Because most patients are prescribed heart rate modulating pharmacotherapy after MI and to maximize generalizability and real-world applicability, the intensity of HIIT was prescribed using RPE. These patients began with four high-intensity intervals of 30–60 s at an RPE of 15–17 interspersed with 1–5 min of low-intensity intervals at an RPE < 14, progressing to 5–8 high-intensity intervals of 2–4 min during 20–45 min of training according to the American College of Sports Medicine (ACSM) guidelines [11]. HIIT was performed only during supervised sessions with frequency not exceeding 3 days/week (non-consecutive days). MICT was performed for 20–45 min at an RPE of 12–14. Heart rate (HR) and rhythm were continuously monitored via a 3-lead electrocardiogram (Q-Tel telemonitoring system, Welch Allyn, USA) with blood pressure (systolic [SBP] and diastolic [DBP]) measured by manual sphygmomanometry. Heart rate (HR) data were retrieved from the exercise session report of each patient, which provided documentation on HRs at specific time-points including: (a) before and after each exercise training session for both HIIT and MICT groups, (b) at the end of the second and third intervals for HIIT, and (c) at the mid-point and end of exercise for MICT. Average HR was calculated by averaging HR values that were documented during exercise. The average metabolic equivalent (MET) and exercise time for each exercise session were reported by the Q-Tel telemonitoring system and documented in exercise session reports. Energy expenditure (EE) was calculated according to the equation: calories = [METs × body weight in kilograms × 3.5]/200 × exercise time, as described previously [12].

### Metabolic syndrome criteria

The definition of metabolic syndrome (MetS) is based on the revised National Cholesterol Education Program’s Adult Treatment Panel III (ATP III) [1]. An ATP III MetS score was calculated as the sum of the ATP III MetS criteria at baseline and completion of CR.

### Metabolic syndrome z-score

Metabolic syndrome z-score is a continuous score of each MetS variable defined by ATP III, and was calculated from modified z-scores of the following variables: [13] waist circumference; SBP; DBP; triglycerides (TG); fasting blood glucose (FBG); ATP III criteria, and standard deviations (denominator of each factor in the formula) using baseline data from the entire study cohort (N = 56): Metabolic syndrome z-score = [(50-HDL)/9.9] + [(TG-150)/69.8] + [(FBG-100)/24.5] + [(WC-88)/10.3] + [(SBP-130)/16.0] + [(DBP-85)/11.2].

### Anthropometric measurement

Dual-energy X-ray absorptiometry (Lunar iDXA Series X, Madison, WI, USA) was conducted by trained radiology technicians using standardized procedures as described previously [14]. The scans were performed within 1 week prior to starting and 1 week after completion of CR. Scans were used to measure total body mass, body fat mass, body lean mass, body fat percentage, and abdominal region fat percentage (the area between the ribs and pelvis). Percentages of the total were calculated accordingly. The scanner was calibrated daily against a manufacturer supplied standard calibration block to control for possible baseline drift. Height was measured using a stadiometer.

### Dietary assessment

Dietary assessment was performed using the Rate-Your-Plate (RYP) questionnaire as previously described [15] during the first and last CR session.

### Statin therapy intensity assessment

Due to the known influence of statin therapy on blood lipids and body composition, we retrieved and analyzed statin therapy intensity data for each patient. Statin therapy intensity was assessed within 1 week prior to the start and within 1 week after the completion of CR and stratified using ACC/AHA guidelines [16]. Statin intensity therapy is defined as a pseudo-continuous variable, 3 = high intensity, 2 = moderate intensity, 1 = low intensity, and 0 = none.

### Exercise capacity

Exercise capacity was evaluated at the beginning and completion of 36 sessions of CR via standard exercise stress test, cardiopulmonary exercise test (CPET) or 6 min walk test (6 MWT). The protocols of pre- and post-intervention were the same for each patient. In the present study, exercise capacity was represented as peak oxygen consumed (VO_2_peak, ml/kg/min) that was directly measured by performing CPET (HIIT = 28; MICT = 9), the VO_2_peak calculated by the equation VO_2_peak = 3.5 ml/kg/min * peak metabolic equivalents (METs) that was determined by standard exercise stress test (HIIT = 8; MICT = 3), or the VO_2_peak calculated from the equation VO_2_peak = 4.9486 + 0.023 * walk distance (meters) that was determined via using 6 MWT (HIIT = 6; MICT = 2) as described previously [17].

### Depressive symptoms assessment

To determine if there was a difference in HIIT- and MICT-induced psychological response, we retrieved Patient Health Questionnaire 9 (PHQ-9) data that was collected monthly for patients to assess patient’s depressive symptoms. The PHQ-9 test was conducted as described previously [18].

### Statistical analysis

The primary outcomes of this study were the mean differences in metabolic syndrome variables from baseline through 36 sessions of CR between groups. A secondary outcome of interest was the mean difference in body composition, including total body fat mass, body fat percentage, abdominal fat percentage, and body lean mass between groups.

Baseline data are presented as frequency and percentage for categorical variables and compared between groups using the Pearson Chi-square test or Fisher exact test. Continuous data are presented as mean and standard deviation and compared between groups using two-sample t-test or non-parametric Wilcoxon rank-sum test, as appropriate depending on the distribution. Prior to analyses, normality was evaluated and the distribution of changes looked approximately normal. Analysis of covariance (ANCOVA) was used to compare the change in each parameter of interest between groups. In this analysis, change was modeled as the outcome in a linear regression model and group effects were tested with adjustment for the baseline value of the parameter being tested. These analyses were repeated in a matched subset of each group to control for potential differences in exercise duration, exercise intensity, and exercise energy expenditure. In order to assess differences over time between groups, a repeated measures analysis of variance (ANOVA) was used. In this analysis, measurements at each week were used to examine the interaction of time and group and tested the differences in effects over time between groups. Energy expenditure and METs were log transformed in these analyses to approximate normality prior to analysis. Means and confidence intervals were back-transformed to the original scale for presentation. Comparisons at each week used the method of Scheffe to adjust P-values for multiple comparisons. SAS version 9.4 (Cary, NC) was used for analyses and Two-sided P-values ≤ .05 were considered to be statistically significant.

## Results

### Study patients

Baseline characteristics were not different between groups (Table 1): age (69 ± 14 vs. 68 ± 10 years *P *= .78), female (36% vs. 36%, *P *> .99), body weight (91.2 ± 14.4 vs. 91.5 ± 16.3 kg *P *= .95), height (170 ± 10 vs. 171 ± 9 cm *P *= .84), and BMI (31.5 ± 4.2 vs. 31.3 ± 4.7 kg/m^2^ *P *= .90). Comorbidities and medications were also not different between groups (*P* > .05). Percentage of PCI (50% vs. 57%) and CABG (14% vs. 14%) were not different between groups (*P* = .64, *P *> .99 respectively). Twenty-five patients suffered one-vessel disease (MICT = 6, 42%; HIIT = 17, 40%; *P *= .88), 17 patients suffered two-vessel disease (MICT = 4, 29%; HIIT = 12, 31%; *P *= .99), 14 patients suffered three-vessel disease (MICT = 4, 29%; HIIT = 13, 31%; *P *= .74). The number of days between patients’ MI event and the beginning of CR was also not different between groups (14 ± 4 vs. 14 ± 11 days, *P* = .34). The VO_2_peak at baseline was not significantly different between groups (23.7 ± 7.1 vs. 23.0 ± 6.3 ml/kg/min, *P *= .73).

**Table 1:** Baseline characteristics of study patients

	MICT (n = 14)	HIIT (n = 42)	*P* value
Age, mean (SD), years	69 ± 14	68 ± 10	*P *= .78
Female sex, n (%)	5 (36)	15 (36)	*P *> .99
Height, mean (SD), cm	170 ± 10	171 ± 9	*P *= .84
Body weight, mean (SD), kg	91.2 ± 14.4	91.5 ± 16.3	*P *= .95
BMI, mean (SD), kg/m^2^	31.5 ± 4.2	31.3 ± 4.7	*P *= .90
Days between CR and MI, mean (SD), days	14 ± 4	14 ± 11	*P *= .34
Medical history, n (%)			
PCI	7 (50)	24 (57)	*P *= .64
CABG	2 (14)	6 (14)	*P *> .99
Hyperglycemia	11 (79)	33 (79)	*P *> .99
Hypertension	8 (57)	20 (48)	*P *= .54
Dyslipidemia	14 (100)	39 (92)	*P *= .57
Ever smoker	5 (36)	14 (33)	*P *> .99
Coronary angiography			
One-vessel disease, n (%)	6 (42)	17 (40)	*P *= .88
Two-vessel disease, n (%)	4 (29)	12 (29)	*P *= .99
Three-vessel disease, n (%)	4 (29)	13 (31)	*P *= .74
Medications, n (%)			
Insulin	3 (21)	9 (21)	*P *> .99
Oral hypoglycemic drugs	4 (29)	15 (36)	*P *= .75
Statins	14 (100)	42 (100)	*P *= .99
ACEIs/ARBs	9 (64)	24 (57)	*P *= .64
Antiplatelet drugs	12 (86)	41 (98)	*P *= .15
Anticoagulants	7 (50)	26 (62)	*P *= .43
Beta receptor blockers	9 (64)	37 (88)	*P *= .10
CCBs	2 (14)	7 (17)	*P *> .99
Diuretics	2 (14)	1 (2)	*P *= .15
Nitrates	4 (29)	6 (14)	*P *= .25

*ACEIs* angiotensin-converting enzyme inhibitors, *ARBs* angiotensin II receptor blockers, *BMI* body mass index, *CABG* coronary artery bypass graft, *CCBs* calcium channel blockers, *HIIT* high-intensity interval training, *MICT* moderate-intensity continuous training, *PCI* percutaneous coronary intervention, *VO*_*2*_*peak* peak oxygen consumed

### Exercise parameters over the course of cardiac rehabilitation

**Fig. 2:**
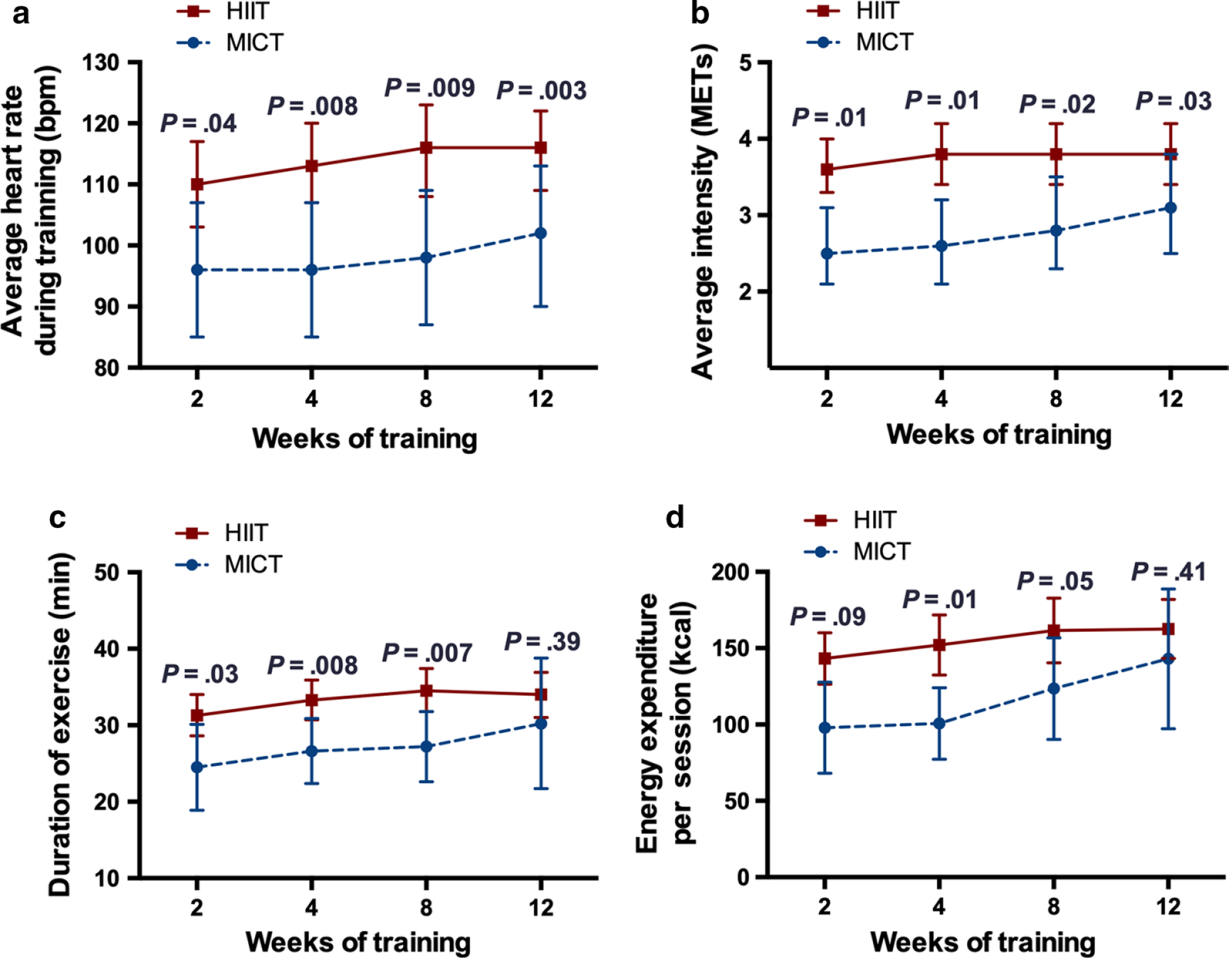
Exercise parameters over the course of cardiac rehabilitation. *HIIT* high-intensity interval training, *MICT* moderate-intensity continuous training. **a** Average heart rate during training. HIIT vs. MICT, 2-weeks: 110 vs. 96 bpm, *P *= .04; 4-weeks: 113 vs. 96 bpm, *P *= .008; 8-weeks: 116 vs. 98 bpm, *P *= .009; 12-weeks: 116 vs. 102 bpm, *P *= .003, respectively. There was no evidence of a difference across groups over time (*P *= .81). **b** Average intensity. 2-weeks: 3.2 vs. 2.6 METs, *P *= .01; 4-weeks: 3.3 vs. 2.5 METs, *P *= .01; 8-weeks: 3.5 vs. 2.9 METs, *P *= .02; 12-weeks: 3.8 vs. 3.2 METs, *P *= .03, respectively. Evidence of a difference across groups over time was noted (*P *= .40). **c** Duration of exercise. 2-weeks: 31 vs. 25 min, *P *= .03; 4-weeks: 33 vs. 27 min, *P *= .008; 8-weeks: 35 vs. 27 min, *P *= .007; 12-weeks: 34 vs. 30 min, *P *= .39, respectively. There was no evidence of a difference across groups over time (*P *= .68). **d** Energy expenditure per session. 2-weeks: 143 vs. 98 kcal, *P *= .09; 4-weeks: 152 vs. 100 kcal, *P *= .01; 8-weeks: 161 vs. 124 kcal, *P *= .05; 12-weeks: 163 vs. 143 kcal, *P *= .41, respectively. There was no evidence of a difference across groups over time (*P *= .37). Results presented are mean and 95% confidence limits based on repeated measures ANOVA. Comparisons at each time point are adjusted for multiple comparisons using the method of Scheffe

The HIIT group demonstrated significantly higher average HR, average exercise intensity, longer duration of exercise, and higher energy expenditure per session at each time point compared to MICT group (*P *< .05), while the average exercise intensity (*P *= .40), duration of exercise at 12-weeks (*P *= .39), and energy expenditure at 2-weeks (*P *= .09) and 12-weeks (*P *= .37) were not different between groups. Further, there were no differences between groups in average HR (*P *= .81), average exercise intensity (*P *= .40), duration of exercise (*P *= .68), energy expenditure per session (*P *= .37) across groups over time (Fig. 2).

### Dietary quality and depressive symptoms assessment

The baseline RYP scores in MICT and HIIT were not different (56 ± 7 vs. 56 ± 7, *P *= .99). The changes in RYP scores between groups were not different from baseline to the completion of 36 CR sessions (5 ± 5 vs. 5 ± 5, P = .46). The changes in PHQ-9 scores were not different across the groups after the completion of 36 CR sessions (− 1.2 ± 3.6 vs. − 1.6 ± 3.5, P = .45).

### Statin therapy intensity assessment

All 56 patients were taking statin therapy at baseline. The changes in average statin therapy intensities between the groups were not different from baseline to the completion of 36 CR sessions (0.1 ± 0.3 vs. − 0.1 ± 0.5, *P *= .30).

### Metabolic syndrome

Compared to MICT, the HIIT group demonstrated greater reductions in MetS presence (relative risk = 0.5, 95% confidence interval 0.33–0.75, *P *< .001), MetS z-score (− 3.6 ± 2.9 vs. − 0.8 ± 3.8, *P *< .001), ATP III MetS scores (*P *= .005) and improvements in individual MetS variables, including, waist circumference (*P *= .01), FBG (*P *< .001), TG (*P *= .001) and DBP (*P *= .001). The changes in HDL-C and SBP were not different between groups (Table 2).

**Table 2:** Outcomes

	MICT (n = 14)		HIIT (n = 42)		ANCOVA
	Baseline	Change	Baseline	Change	P-value
Rate-Your-Plate	56 ± 7	5 ± 5	56 ± 7	5 ± 5	*P *= .46
Average statin	2.4 ± 0.7	0.1 ± 0.3	2.8 ± 0.5	− 0.1 ± 0.5	*P *= .30
MetS variables					
MetS z-score	5.7 ± 3.6	− 0.8 ± 3.8	4.6 ± 3.4	− 3.6 ± 2.9	*P *< .001
ATP III MetS score	3.9 ± 0.7	− 0.6 ± 1.0	3.6 ± 0.6	− 1.2 ± 1.0	*P *= .005
Waist circumference, cm	110 ± 11	1 ± 5	110 ± 10	− 3 ± 5	*P *= .01
FBG, mg/dl	130.4 ± 38.7	− 3.9 ± 25.8	129.3 ± 37.3	− 25.8 ± 34.8	*P *< .001
TG, mg/dl	222.5 ± 80.1	− 10.4 ± 105.3	185.5 ± 101.5	− 67.8 ± 86.7	*P *< .001
HDL-c, mg/dl	38.9 ± 10.8	6.4 ± 8.3	40.3 ± 9.8	3.8 ± 9.0	*P *= .42
SBP, mmHg	122 ± 18	0 ± 25	122 ± 15	− 6 ± 18	*P *= .16
DBP, mmHg	71 ± 12	0 ± 13	68 ± 11	− 7 ± 11	*P *= .001
Body composition					
Body weight, kg	91.2 ± 14.4	0.6 ± 4.8	91.5 ± 16.3	− 0.5 ± 4.9	*P *= .45
BMI, kg/m^2^	31.5 ± 4.2	0 ± 1.5	31.3 ± 4.7	− 0.1 ± 1.7	*P *= .83
Fat mass, kg	35.4 ± 6.6	0 ± 2.2	35.7 ± 8.5	− 2.1 ± 2.1	*P *= .002
Body fat, %	40.2 ± 4.9	− 0.2 ± 1.4	39.9 ± 5.9	− 1.8 ± 1.7	*P *= .002
Lean mass, kg	53.5 ± 9.3	− 0.9 ± 3.2	53.5 ± 9.8	0.9 ± 1.9	*P *= .01
Abdominal fat, %	47.5 ± 5.2	0.1 ± 2.4	49.6 ± 5.7	− 2.2 ± 2.6	*P *= .004
Peak VO_2_, ml/kg/min	23.7 ± 7.1	1.4 ± 0.9	23.0 ± 6.3	5.0 ± 2.5	*P *= .03
PHQ-9	4.0 ± 2.1	− 1.2 ± 3.6	4.0 ± 3.2	− 1.6 ± 3.5	*P *= .45

*BMI* body mass index, *CR* cardiac rehabilitation, *DBP* diastolic blood pressure, *FBG* fasting blood glucose, *HDL-c* high-density lipoprotein cholesterol, *HIIT* high-intensity interval training, *MetS* metabolic syndrome, *MICT* moderate-intensity continuous training, *Peak VO*_*2*_ peak oxygen consumed, *PHQ-9* patient health questionnaire 9, *SBP* systolic blood pressure, *TG* triglycerides

### Body composition

**Fig. 3:**
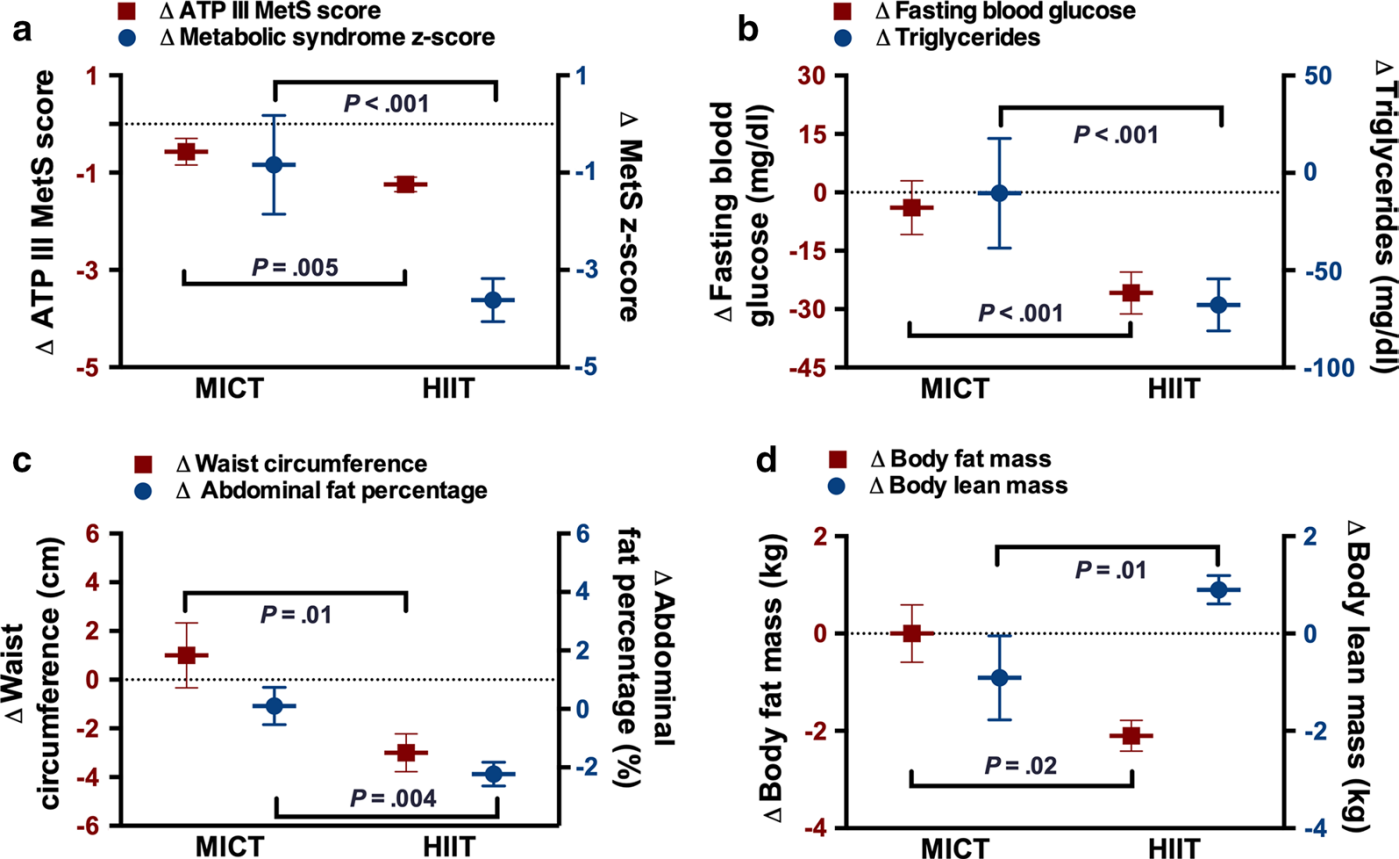
Changes in metabolic syndrome and body composition variables from the baseline to the completion of 36 sessions of cardiac rehabilitation across MICT and HIIT groups. *ATP III* National Cholesterol Education Program’s Adult Treatment Panel III, *HIIT* high-intensity interval training, *MetS* metabolic syndrome, *MICT* moderate-intensity continuous training. Values are reported as mean ± standard deviation

The HIIT group demonstrated greater improvements in total body fat mass (*P *= .002), body fat percentage (*P *= .002), abdominal fat percentage (*P *= .004), and body lean mass (*P *= .01) compared to MICT (Fig. 3).

### Sub-group analysis

Due to the difference in exercise energy expenditure between HIIT and MICT, we matched HIIT and MICT participants for exercise energy expenditure. In this sub-group analysis, the HIIT group demonstrated greater improvements in MetS z-score (*P *< .001), ATP III MetS score (*P *= .005), and MetS variables (including FBG (*P *= .002), TG (*P *= .008), DBP (*P *< .001), total body fat mass (*P *= .02), body fat percentage (*P *= .01), body lean mass (*P *= .03), and abdominal fat percentage (*P *= .01) compared to MICT, with no differences in RYP scores (*P *= .95) and average statin therapy intensity between groups from baseline to follow-up (*P *= .23) (Table 3).

**Table 3:** Outcomes after matching for differences in exercise energy expenditure

	MICT (n = 14)		HIIT (n = 14)		ANCOVA
	Baseline	Change	Baseline	Change	P-value
Rate-Your-Plate	56 ± 7	5 ± 5	55 ± 7	5 ± 7	*P *= .95
Average statin	2.4 ± 0.7	0.1 ± 0.3	2.6 ± 0.7	− 0.1 ± 0.3	*P *= .23
MetS variables					
MetS z-score	5.7 ± 3.6	− 0.8 ± 3.8	5.6 ± 3.2	− 5.3 ± 3.0	*P *< .001
ATP III MetS score	4 ± 1	− 1 ± 1	4 ± 0	− 2 ± 1	*P *= .005
Waist circumference, cm	110 ± 11	1 ± 5	103 ± 9	− 0.4 ± 2.2	*P *= .31
FBG, mg/dl	130.4 ± 38.7	− 3.9 ± 25.8	138.9 ± 46.6	− 40.1 ± 46.6	*P *= .002
TG, mg/dl	222.5 ± 80.1	− 10.4 ± 105.3	232.0 ± 111.1	− 97.2 ± 105.7	*P *= .008
HDL-c, mg/dl	38.9 ± 10.8	6.4 ± 8.3	37.6 ± 7.3	3.7 ± 4.8	*P *= .26
SBP, mmHg	122 ± 18	0 ± 25	122 ± 15	− 13 ± 12	*P *= .13
DBP, mmHg	71 ± 12	0 ± 13	70 ± 7	− 12 ± 8	*P *< .001
Body composition					
Body weight, kg	91.2 ± 14.4	0.6 ± 4.8	80.3 ± 13.2	1.4 ± 7.0	*P *= .72
BMI, kg/m^2^	31.5 ± 4.2	− 0.1 ± 1.5	28.6 ± 4.5	0.7 ± 2.4	*P *= .92
Fat mass, kg	35.4 ± 6.6	0 ± 2.2	30.9 ± 7.6	− 1.7 ± 1.8	*P *= .02
Body fat, %	40.2 ± 4.9	− 0.2 ± 1.4	38.8 ± 7.5	− 1.8 ± 1.7	*P *= .01
Lean mass, kg	53.5 ± 9.3	− 0.9 ± 3.2	48.5 ± 8.5	1.2 ± 1.4	*P *= .03
Abdominal fat, %	47.5 ± 5.2	0.1 ± 2.4	48.1 ± 7.4	− 2.4 ± 2.4	*P *= .01

*BMI* body mass index, *CR* cardiac rehabilitation, *DBP* diastolic blood pressure, *FBG* fasting blood glucose, *HDL-c* high-density lipoprotein cholesterol, *HIIT* high-intensity interval training, *MetS* metabolic syndrome, *MICT* moderate-intensity continuous training, *SBP* systolic blood pressure, *TG* triglycerides

## Discussion

This study has, for the first time, demonstrated that supervised HIIT elicited greater improvements in MetS z-score and ATP III MetS score, and some components of MetS that include waist circumference, FBG, TG, and DBP, than MICT in MI patients with MetS enrolled in early outpatient CR. These findings support the use of HIIT, as an alternative and/or complimentary to MICT, for MI patients with MetS to improve CVD risk factor related outcomes.

Our findings demonstrate that HIIT decreased the prevalence of MetS and MetS severity (MetS z-score) to a greater extent than MICT in MI patients enrolled in early outpatient CR. The observed magnitude of HIIT-induced benefit in the current study parallels a recent study by Morales-Palomo et al. [19] in individuals with MetS but without MI. These authors found that 2 yearly 16-week HIIT programs resulted in chronically lower MetS and halted the increase in medication use that occurs in non-exercising MetS individuals. Similarly, Ramos et al. [8] demonstrated low-volume HIIT was sufficient to ameliorate the MetS severity in middle-aged adults, and was superior to MICT in terms of time efficiency. In addition, an across race study demonstrated that high-intensity interval and resistance training led to improvements in cardiometabolic risk factors, including FBG, TG, and SBP, in both Mapuche and European hyperglycemic adult women [20]. Our findings are consistent with these observations further supporting the important impact of HIIT on components of MetS and suggest that HIIT could be encouraged for MI patient with MetS.

The present study, for the first time, demonstrated that HIIT contributes to greater decreases in total body fat mass (*P *= .002), body fat percentage (*P *= .002), and abdominal fat percentage (*P *< .004) while increasing body lean mass (*P *= .01) in MI patients with MetS compared to MICT. Several other studies have also examined the efficacy of HIIT on reducing subcutaneous and abdominal fat in overweight adolescents [21] and type 2 diabetic men [22]. Trapp et al. [23] compared HIIT and MICT in young women and found that HIIT resulted in greater reductions in subcutaneous fat. These are important findings, as increasing evidence suggests body fat (and its distribution) and lean body mass are associated with risk of incident MetS [24, 25]. For example, risk of MetS for individuals in the highest abdominal fat quintile is more than three times greater compared with the lowest quintile [25]. Moreover, individuals with high fat and low muscle mass have an approximately twofold increase in the risk of developing MetS [24]. We have also demonstrated that total body fat mass, abdominal fat percentage, and body lean mass are independent risk factors for long-term cardiovascular events and mortality in patients with [26, 27] and without coronary heart disease (CHD) [28]. In patients with CHD in the highest quartile of body fat percentage, risk of cardiovascular events is double compared to the lowest quartile [27]. In contrast, two recent studies by Keating et al. [29] and Wewege et al. [30] demonstrated that HIIT and MICT elicited similar benefits for improvement in body composition for overweight and obese adults, especially once controlled for energy expenditure. Our findings combined with others support that HIIT may provide similar or enhanced benefits for risk factor management and should be viewed as an important treatment strategy for MI patients with MetS.

This study showed that there were no changes in body mass and BMI in either MICT or HIIT groups, which are consistent with previous studies by Keating et al. [29] and Wewege et al. [30] that demonstrated that HIIT and MICT elicit similar changes in body mass. This phenomenon may be related to the obesity paradox. Overweight and obesity are associated with an increased likelihood of developing cardiovascular disease and all-cause mortality [31] and have become increasingly common worldwide, with overweight and obesity conventionally defined as a BMI of > 25 kg/m^2^ and > 30 kg/m^2^, respectively. In contrast to the general population, patients with CHD demonstrate an inverse relationship between BMI and mortality, termed the obesity paradox [32]. However, it is now apparent that this paradox is related to the preservation of lean muscle mass as there is no paradox in the general population [33] or patients with CHD [27] when measuring body fat percentage instead of BMI. This point helps to understand how MetS parameters were improved in our HIIT patients, even in the absence of BMI changes.

A variety of HIIT protocols have been developed and used for CHD patients [34], which vary in intensity, interval duration, and the number of intervals. Further, exercise intensity is generally prescribed using a percent of maximal oxygen consumption, percent of maximal heart rate, percent of maximal power, or RPE (rated perceived exertion, Borg scale) [34, 35]. The present study used RPE to prescribe the intensity of exercise with accompanying HR monitoring. HIIT prescribed using RPE has been used across the aging spectrum [36, 37]. Buchheit et al. [38] and Levinger et al. [39] demonstrated that the RPE has shown a great correlation with HR, ventilation, and VO_2_ in individuals both with and without CAD, and the correlation is not impacted by beta-blocker medication, a commonly used HR modulating medication by patients with MI [39]. In addition, HIIT prescribed by RPE can promote greater positive responses than both HIIT and MICT when prescribed with imposed intensity, such as workload and HR, due to the perceived autonomy, time efficiency, and changing stimulus [36]. The present study demonstrates that RPE should be considered an appropriate alternative or supplementary method to HR for prescribing exercise intensity for HIIT in MI patients with MetS.

To determine the relation of energy intake, statin therapy intensity, and energy expenditure (EE) during exercise and HIIT-induced improvements in MetS and body composition, the present study assessed dietary quality, average statin therapy intensity and the EE per exercise session over the course of 36 sessions of CR. Our results showed that there were no differences in RYP scores (dietary quality) and statin therapy intensity across groups from baseline to follow-up compared to MICT. Additionally, HIIT induced similar exercise EEs to MICT at the beginning and end of 36 CR sessions. These findings suggest that energy intake, statin therapy intensity, and EE during exercise are not primary explanations for the HIIT-induced greater improvements in MetS and body composition than MICT in these patients. To confirm the accuracy of these findings, we matched a sub-group of HIIT and MICT patients on exercise EE specifically and found that the improvements in MetS and body composition in the HIIT group persisted.

The exact mechanisms whereby HIIT leads to greater improvements in MetS variables and body composition in MI patients with MetS remain unclear. The mechanisms may involve a wide range of systems responsible for the physiologic adaptations to structured exercise, including the cardiovascular system, respiratory system, and musculoskeletal systems. Increasing evidence suggests that HIIT can improve post-infarction left ventricular remodeling [40], augment stroke volume in patients with type 2 diabetes [41], optimize heart rate recovery after exercise in MI patients [42], and improve brachial artery flow-mediated dilation in sedentary adults [43] and type 2 diabetes [44, 45], which together may contribute to the improvements in insulin sensitivity, blood pressure, and body composition [46]. With regards to the respiratory system, respiratory muscle dysfunction is a common manifestation in older patients with CVD [47], Tasoulis et al. demonstrated that 12 weeks of HIIT significantly improved respiratory muscle function in older adults with HF [48]. With respect to skeletal muscle, we have previously shown that 4-weeks of aerobic exercise can enhance skeletal muscle mitochondrial biogenesis and function in male mice [49]. Motta et al. [50] also showed that HIIT ameliorated fructose-induced metabolic dysfunction in male mice through mitochondrial biogenesis and β-oxidation, via irisin and PGC1 α in skeletal muscle. Moreover, several recent trials have demonstrated that 3–12 weeks of HIIT can significantly increase resting metabolic rate [51] and post-exercise oxygen consumption in healthy adults [52], and fat oxidation in obese adolescents [53]. In summary, HIIT can elicit numerous improvements in organ structure and physiological functions of multiple systems; however, more research is warranted to better understand the primary mechanisms of HIIT-induced cardiometabolic benefits.

With respect to the safety of performing HIIT, all participants enrolled in this study completed 36 sessions of CR without a single major adverse event. Although no major adverse events were registered, the current study was not designed/powered to demonstrate safety of HIIT in patients with MI. However, the safety of supervised HIIT in CR is generally well documented in patients with established coronary artery disease [54, 55].

## Study limitations

It is important to recognize the retrospective, observational nature of the present study design. Additional research in this area is encouraged to confirm these findings in a prospective fashion. To avoid bias associated with the number of HIIT sessions completed, we only enrolled the patients who completed the standard 36 sessions of outpatient CR and attended follow-up assessments, which may have introduced selection bias. The patients in this study are overweight; therefore, additional research focusing on MI patients with MetS within “normal weight” is encouraged. Furthermore, although not unusual in similar studies, an unintended consequence of our clinical population is that only 36% were women. Thus, additional studies may be needed to ensure the generalizability of our findings.

## Conclusions

The present study demonstrates that supervised HIIT prescribed by RPE can elicit greater improvements in MetS and body composition for MI patients with MetS compared to MICT. These findings support the use of HIIT as a treatment tool to improve MetS components in MI patients enrolled in early outpatient cardiac rehabilitation.

## Data Availability

Not applicable.

## Abbreviations

MetS: metabolic syndrome
MI: myocardial infarction
HIIT: high-intensity interval training
MICT: moderate-intensity continuous training
CR: cardiac rehabilitation
DXA: dual-energy x-ray absorptiometry
RPE: rating of perceived exertion
ACSM: American College of Sports Medicine
HR: heart rate
SBP: systolic blood pressure
DBP: diastolic blood pressure
ATP III: Adult Treatment Panel III
TG: triglycerides
FBG: fasting blood glucose
HDL: high density lipoprotein
RYP: rate your plate
CPET: cardiopulmonary exercise test
6 MWT: 6 min walk test
VO_2_peak: maximal oxygen consumption
ANCOVA: analysis of covariance
ANOVA: analysis of variance
CHD: coronary heart disease
EE: energy expenditure
